# Lumbar Schmorl's Nodes and Their Correlation with Spine Configuration and Degeneration

**DOI:** 10.1155/2018/1574020

**Published:** 2018-11-07

**Authors:** Janan Abbas, Kamal Hamoud, Natan Peled, Israel Hershkovitz

**Affiliations:** ^1^Department of Anatomy and Anthropology, Sackler Faculty of Medicine, Tel Aviv University, Tel Aviv 6997801, Israel; ^2^Department of Physical Therapy, Zefat Academic College, Zefat 13206, Israel; ^3^Faculty of Medicine in the Galilee, Bar-Ilan University, Zefat 1311502, Israel; ^4^Department of Orthopaedic Surgery, The Baruch Padeh Poriya Medical Center, Tiberias 1520800, Israel; ^5^Department of Radiology, Carmel Medical Center, Haifa 3436212, Israel

## Abstract

The aim of this study was to reveal whether demographic aspect, vertebral morphometry, and spine degeneration are associated with lumbar Schmorl's nodes (SNs). A retrospective cross-sectional study was performed using data from the Department of Radiology (Carmel, Medical Center, Israel) for 180 individuals: age range between 40 and 99 years; 90 males and 90 females. All participants had undergone high-resolution CT scans for abdominal diagnostic purposes in the same supine position prior to our study, which enabled the processing of the scans in all planes and allowed a 3D reconstruction of the lower lumbar region. Eighty individuals (44.4%) had at least one SN along the lumbar spine, particularly at L3-4 level (30%). Vertebral body length (L1 to L3) and width (L1 and L4) were significantly greater in the SNs group compared to non-SNs group. On contrast, disc height (L3-4 and L4-5) was significantly lesser in SNs group than non-SNs group. SNs was significantly associated with smoking (*X*^2^= 4.436, P=0.02) and degenerative lumbar spinal stenosis (*X*^2^= 5.197, P=0.038). Moreover, the prevalence of SN was significantly greater in individuals with vacuum phenomenon and osteophytes formation (L1-2 to L4-5 levels). This study indicates that vacuum phenomenon on L3-4 (OR: 4.7, P=0.034), smoking habit (OR: 3.2, P=0.003), disc height loss of L4-5 (OR: 0.798, P=0.008), vertebral body length of L1 (OR: 1.37, P<0.001), and age (OR: 1.05, P=0.002) increase the probability of developing lumbar SNs.

## 1. Introduction

Schmorl's nodes (SNs) have been described as herniation of nucleus material through the endplate into the vertebral body [1, 2]. The nodes appear on computed tomography (CT) scans as a round or sclerotic irregular area of bone density with a sclerotic circumferential margin lying beneath the cartilaginous endplate [3].

SNs can appear on any spine vertebra, mainly in the lower thoracic and lumbar regions [1, 4]. SNs are considered a multifactorial origin and can be associated with trauma to the spine and several diseases such as osteoporosis and metabolic disease [1, 4, 5]. The reported frequency of SNs in the general population varies from 2 to 76% [6–10] with considerable preference for males [4, 6, 11].

Although the etiology of SNs remains elusive, both congenital [6, 12, 13] and traumatic [13–15] factors have been suggested. SNs were found to be associated with disc degeneration [6, 16, 17] and back pain [5, 14, 18], while their association with vascular disease (e.g., diabetic mellitus) and occupational stress is less clear. Previous skeletal studies [19, 20] have noted a correlation between SNs and vertebra size and shape. To our knowledge, no study to date has investigated the correlation between SNs and degenerative changes on the posterior spine element (e.g., facet-joint arthrosis).

The aims of this study were (1) to reveal the association between SNs and demographic factors (e.g., occupation stress and diabetes mellitus), (2) to examine the relationships between vertebral morphometry (e.g., vertebral body width and length) and SNs, and (3) to determine whether the presence of SNs in the lumbar spine accelerates a degenerative process of the spine elements.

## 2. Materials and Methods

### 2.1. Study Design

This cross-sectional retrospective study was performed on 180 adult individuals (mean age = 62.5 years; range: 40-99 years), 90 males and 90 females, having undergone high-resolution CT scans (Brilliance 64, Philips Medical Systems, Cleveland, OH; slice thickness 0.9–3mm, voltage 120 kV, current 150–570 mA) for abdominal diagnostic purposes prior to our study at the Department of Radiology (Carmel Medical Center, Haifa, Israel). All CT images were done in a supine position with extended knee.

We used the CT scan (‘‘Extended Brilliance Workspace” portal) that enabled the processing of the scans in all planes and allowed a 3D reconstruction of the lower lumbar region. Analysis of all the CT scans was performed by the first author (JA) under the supervision of a diagnostic radiologist (NP, Head of the Department of Radiology, Carmel Medical Center and Head of Israeli National Council for Medical Imaging).

This study was approved by the Ethics Committee of the Carmel Medical Center (0083-07-CMC).

### 2.2. Identification of SNs

**Figure 1:**
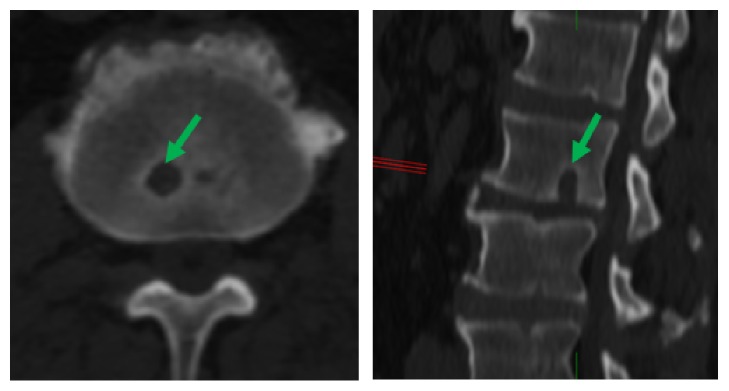
Schmorl's node identification in axial (left) and sagittal (right) planes.

Schmorl's node is defined as a focal lesion in the vertebral endplate usually with sclerotic margins. A lesion with a depth of ≥ 2 mm was considered in the current study. Both axial and sagittal planes were utilized for this purpose (Figure 1). The presence of Schmorl's nodes on the cranial and caudal endplate surfaces at the lumbosacral region (from L1 to S1 vertebra) was recorded.

The study population was divided into 2 main groups: without SN (1) and with SN (2); the latter included all individuals who had at least 1 SN. For further statistical analysis we defined a third group (multi-SN) that included all subjects who had at least two affected surfaces along the lumbar spine with SN.

### 2.3. Demographic and Health Aspects

The demographic data (e.g., BMI, occupation, and number of deliveries) and vascular diseases (hypertension and diabetes mellitus) for all the participants were obtained from both the interviews and medical records. Occupation was divided into four categories: (a) heavy manual labor, (b) housekeeping, (c) work requiring prolonged sitting, and (d) other. All participants were also classified as smokers (≥ 10 cigarettes per day for at least five years) or nonsmokers. In order to identify the association between SNs and age, we classified the participants into two age groups: (a) middle group included individuals between 40 and 60 years and (b) older group included individuals who were 60 years and over.

### 2.4. Spine Configuration

*Vertebral body width (VW) and length (VL)* were measured in the axial plane at the mid-vertebral height.

#### 2.4.1. Bony Canal Diameters

Anterior-posterior (AP) and mediolateral (ML) diameters were measured in the axial plane at the mid-vertebral body, at the level of the basivertebral foramen.

#### 2.4.2. 

*Lumbar lordosis* angle was measured in the sagittal, at the meeting point between a line running parallel to the upper discal surface of L1 and a line running parallel to discal surface of S1.

### 2.5. Spinal Disorder and Degeneration

#### 2.5.1. 

*Lumbosacral transitional vertebra (LSTV)* was recorded following the classification of Castellvi et al. (1984) and defined as a total or partial unilateral fusion of the transverse process of the lowest lumbar vertebra to the sacrum [21].

#### 2.5.2. 

*Osteophyte formation* was evaluated in the axial CT images and recorded for each vertebral body at the superior and inferior endplates. In order to determine the degree of osteophyte by lumbar level, we classified the osteophytes' formation into three groups: (1) no osteophytes, (2) individuals who manifested osteophyte at the margin of one discal surface of a vertebra, and (3) individuals who had osteophyte on both discal surfaces (superior and inferior).

#### 2.5.3. 

*“Vacuum phenomenon”* is an accumulation of gas, appearing as a “black spot” in the intervertebral disc, and was evaluated in the axial plane at the level of the intervertebral disc.

#### 2.5.4. 

*Disc herniation* was observed in both axial and sagittal planes following the method of Jensen et al. [1994] [22].

#### 2.5.5. 

*Intervertebral disc height (IDH)* was measured in the mid-sagittal plane at three points: anterior, middle, and posterior. Mean IDH was then calculated for the three different locations.

#### 2.5.6. Degenerative Spondylolisthesis (DS)

This phenomenon was evaluated in the mid-sagittal plane and considered positive when the amount of listhesis exceeded 3 mm [23, 24].

#### 2.5.7. Facet Joint Arthrosis

This was evaluated according to the method of Pathria et al. (1987) [25]. Facet arthrosis for each level was considered positive if at least one side was affected.

#### 2.5.8. Ligamentum Flavum (LF) Thickness

This was measured in the axial plane, using the method of Fukuyama et al. (1995) [26]. Mean LF was then calculated for right and left sides.

#### 2.5.9. Degenerative Lumbar Spinal Stenosis (DLSS)

This was evaluated in the axial plane by measuring the cross-sectional area (CSA) of the dural sac at the intervertebral disc level. DLSS was defined positive when this measure was ≤ 75 mm^2^ (following Schonstrom et al.) at least on one lumbar level [27].

### 2.6. Statistical Analysis

The statistical analysis was conducted using SPSS v. 20.0. Chi-Square (*X*^2^) or Fisher's exact tests were used to determine the association between SN and categorical parameters. T-test and ANCOVA t-test (adjusted for age) were also taken to identify the correlation between SNs and metric parameters. A Binary Logistic Regression analysis was carried out to allocate the variables that affect the probability of SNs development (dependent variable, SN) using “Forward LR” method. Significant difference was set at P < 0.05.

## 3. Results

The intraclass correlation coefficient (ICC) test for intratester and intertester reliabilities of the parametric variables (e.g., ligamentum flavum thickness and lumbar lordosis) ranged between ICC= 0.866 to 0.970 and ICC= 0.737 to 0.910, respectively. Kappa index for categorical data ranged within K= 0.894 to 0.999 for intraobserver reliabilities and K= 0.780 to 0.984 for interobserver reliabilities.

### 3.1. Prevalence and Demographic and Health Aspects

Of 180 participants, 80 individuals manifested SNs (44.4%); 45 were males (56.2%) and 35 females (43.8%). Multiple nodes were found in 22.2% of the individuals (n=40).

**Figure 2:**
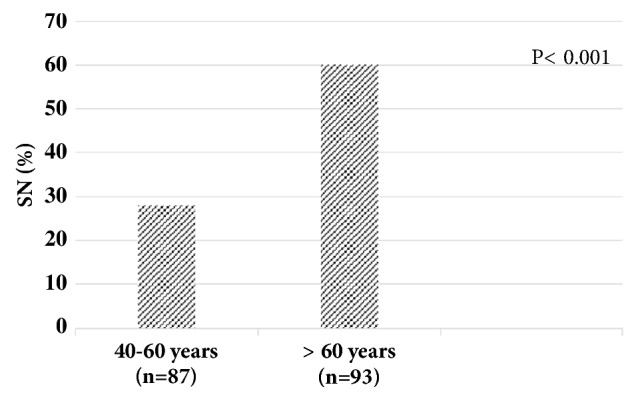
The prevalence of Schmorl's node (SN) in two age groups.

The distribution of SN along the lumbar level was as follows: 22% at L1-2, 21% at L2-3, 30% at L3-4, 18% at L4-5, and 9% at L5-S1. SNs were associated with age; i.e., it was more common in the older age group (Figure 2) (*X*^2^= 19.382, P<0.001), but not with BMI and number of deliveries (P= 0.252 and 0.174, respectively).

Smoking was significantly associated with SNs (*X*^2^= 4.436, P=0.02) (Table 1). No association was found between SNs and vascular disease and/or heavy labor (Table 1). Yet, a significant association was found between multiple SNs and vascular disease (*X*^2^= 4.436, P=0.047).

### 3.2. Spine Configurations

Mean bony canal diameters were similar in the SN and non-SN groups, except for mediolateral diameter at L2 vertebra (P=0.008). Conversely, the vertebral body length (L1 to L3) and width (L1 and L4) were significantly greater in the SN group than in the non-SN group (Table 2). No association between lumbar lordosis and SNs was noted (P=0.845).

### 3.3. Spine Disorder and Degeneration

Neither lumbosacral transitional vertebrae nor degenerative spondylolisthesis was associated with SN (P>0.05).

SN was significantly associated with DLSS development (*X*^2^=5.197, P=0.038).

Disc herniation on L4-5 level was significantly related to SNs (*X*^2^= 8.229, P=0.01). Furthermore, disc height (L3-4 and L4-5) was significantly lower on the level of affected SN compared to healthy one (P<0.05).

**Figure 3:**
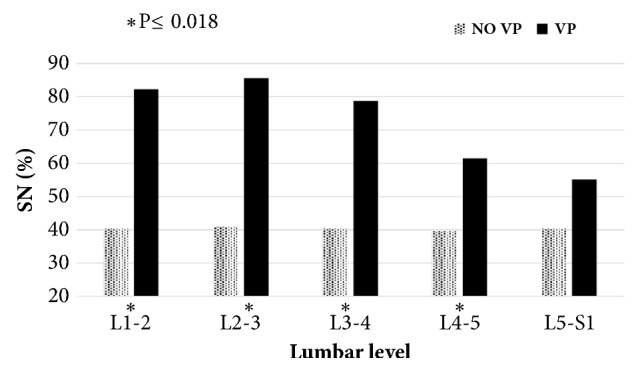
The prevalence of Schmorl's node (SN) in individuals with and without vacuum phenomenon (VP) by lumbar level.

**Figure 4:**
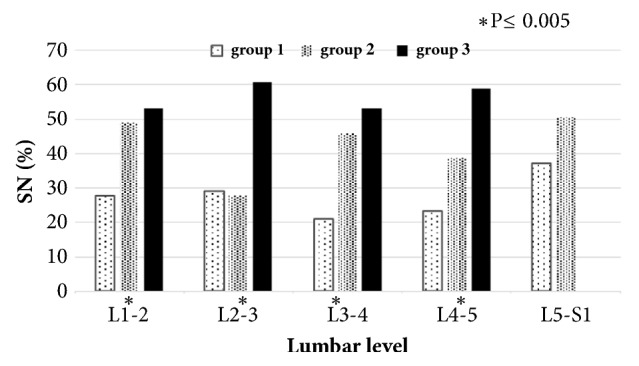
The prevalence of Schmorl's node (SN) in individuals with and without osteophyte formation by lumbar level. Group 1: no osteophytes, group 2: individuals with osteophyte at one discal surface, and group 3: individuals with osteophyte on both discal surfaces.

The prevalence of SN along the lumbar spine was significantly greater in individuals with vacuum phenomenon and osteophytes formation (from L1-2 to L4-5 levels) (Figures 3 and 4).

Individuals with SNs manifested greater ligamentum flavum thickness on L2-3 (2.4 mm ± 0.8 vs. 2.1 mm ± 0.6, P= 0.012) and higher prevalence of facet joint arthrosis on L4-5 (97.5% vs. 88%, P= 0.023) compared to non-SNs group. Nevertheless, no segmental correlation between SNs and either facet joint arthrosis or ligamentum flavum thickness was found on adjacent level (P>0.5).

### 3.4. Logistic Regression

According to our findings, vacuum phenomenon of L3-4 disc (OR: 4.7, P=0.034), smoking (OR: 3.2, P=0.003), disc height of L4-5 (OR: 0.792, P= 0.008), vertebral body length of L1 (OR: 1.37, P< 0.001), and age (OR: 1.05, for each additional year, P=0.002) increase the likelihoods of lumbar SNs.

## 4. Discussion

To the best of our knowledge, this is the first study that has addressed the prevalence and potential causes of SN using CT assessment. Our finding indicates that 44% of the studied individuals presented SN (at one or more lumbar levels). This prevalence falls within the range reported by other studies [2, 5, 8, 10, 11, 16]. More so, this prevalence of SN is considered relatively high compared to the rates obtained from radiological images (3.8%-38% of spine) and almost similar to those reported for skeletal material [2, 5, 10, 28, 29]. This implies that CT images can be confidently used for evaluating SNs in the living populations.

### 4.1. SNs Location

Our finding shows that the majority of SNs presented in the upper lumbar spine region (mainly L3-4 level) rather than the lower one. This outcome could be in agreement with previous reports noting that SNs were commonly located in the lower thoracic and upper lumbar regions [2, 5, 6, 16, 30]. As the majority of these studies reported their SNs prevalence for each vertebra (superior and inferior endplate surfaces) rather than for lumbar level, this research can be compared only with one study [17]. Mok et al. [17] have shown that half of SNs are located in the upper 2 lumbar levels (54.1%) and only 19% are presented on L3-4. Conversely, we found that half (51%) of SNs are presented in the second and third lumbar levels while 30% of those are on L3-4. We believe that this discrepancy could be related to the fact that the mean age of our sample was greater than the latter study (62.5 vs. 40.4 years). Older individuals manifest greater degenerative disc disease (DDD) [31], namely, in the lower lumbar region [32]; therefore, SNs development on this area is expected because it positively correlated with DDD [17]. The higher incidence of SN on L3-4 level could be attributed to the fact that the overall loading onto the spine increases caudally [33]; therefore, the incidence of SN over the lower lumbar region would be the highest due to the overall high mechanical load concentrated over the area. The mechanical strength, however, on both superior and inferior endplates of lumbar region tended to increase caudally, suggesting that the endplates of the upper lumbar segments were weaker than those of the lower segments [34]. We suggest that these facts may explain why the middle part of lumbar region is susceptible to SNs development.

### 4.2. Age

Based on this study, age was significantly associated with SN (OR= 1.05 for each additional year, P=0.002). This result is in agreement with some previous studies [2, 5, 35, 36], but not with another [11]. Wang and colleagues [35] have recently reported a significant association between age and SN (OR= 1.04, P=0.003). In addition, Mok et al. [17] found that mean age of SN was significantly higher than non-SN group; however, an association between age and SN was not discerned. The association between age and SNs is related to the reduction of vertebral body bone density with advanced age. More so, decrease of bone density was evident in the development of irregular Schmorl's nodes [37]. It has been also proposed that degeneration of the cartilaginous endplate due to the aging process produces sites of weakness resulting in SN formation [30]. We believe that this outcome can support the notion that age or any associated factors might play an important role in SN pathogenesis [35].

### 4.3. Smoking and Vascular Disease

Smoking was significantly associated with SN (OR = 3.2, =0.003). In addition, the presence of vascular disease (e.g., diabetic and arterial hypertension) was significantly associated with multiple SNs. The association between SNs and smoking has not sufficiently been addressed in the past. Mok et al. [17] had already mentioned that smoking habit was marginally associated with SN. Furthermore, some papers implicated cigarette smoking as a risk factor for degenerative disc disease [38–41]. The association between smoking and SN could be related to the fact that these nodes precede disc disease [17]. The vasospasm or arteriosclerotic changes caused by smoking may have a negative effect on the blood supply of the vertebral bodies and other structures surrounding the disc, subsequently, affecting disc nutrition [38]. In addition, some of the chemical exposures from cigarette smoke also may have deleterious effects on disc metabolism and accelerate degeneration [38].

Our results indicate that vascular disease is associated with multi-SNs. It has been reported that atherosclerosis, measured as aortic calcifications, was considered an aggravating factor for lumbar disc disease [42]. It was also suggested that diabetes mellitus, which affects the small vessels, causes feeding disturbances to the disc, leading to DD [43].

### 4.4. Vertebral Morphology

Our results indicate that both vertebral body length (L1 to L3) and width (L1, L4) are significantly higher in SN group than those of non-SN group. In addition, vertebral body length of L1 increases the likelihood of SN development (OR=1.37, P<0.001). As there is a lack of relevant data in the literature, this result could not be supported. It was reported, in a medieval and post-medieval skeletons study, that vertebral bodies from lower thoracic region that were affected with SNs are significantly larger than those of a healthy group [19]. It was also noted that individuals with degenerative lumbar stenosis manifest greater lumbar vertebral size [44], and these individuals have higher incidence of SNs compared to the control group [45]. Harrington et al [46] found a positive correlation between lumbar vertebral size and “classical” disc herniation. They suggested that the diameters of the vertebral disc influence its ability to withstand tension during compression according to LaPlace's law [47]. We believe that bigger vertebral body sizes are weaker to resist vertical stress, i.e., SN. This could be supported by the evolutionary theory claiming that greater vertebrae size is associated with reduced bone strength [48].

### 4.5. Spine Degeneration

The results of this study indicate that SNs are significantly associated with disc disease (e.g., disc height loss) and DLSS. The relationship between SNs and DLSS is not surprising as SNs were established to increase the likelihood of symptomatic DLSS [45]. Although the association between SN and disc height loss has been previously reported [16, 17], the precise origin behind this correlation is still debated. Furthermore, VP was found to increase the likelihood of SN development.

Vacuum disc phenomenon refers to the radiographic appearance of gaseous collections in intervertebral disc space, usually in the lumbar region [49–51]. It is produced by the liberation of gas, mostly nitrogen, from surrounding tissues, and accumulation within cracks, clefts, or crevices, which form in the degenerated disc [49, 52–55]. The incidence of discal VP increases with age, and it was reported in approximately 50% of subjects over 40 years of age [56].

The association between disc VP and SNs could be supported by the notion that endplate integrity and function are crucial to the maintenance of mechanical environment and also proper nutrition to the avascular disc [31, 57–64]. It was also proposed that endplate lesion such as SN could accelerate disc degeneration [17, 65]. In contrast, advanced disc disease even for grades 3 and 4 according to Pfirrmann classification [66] was evident for normal endplate [65]. We postulate that the vacuum phenomenon could precede SNs development. Intraspinal gas is associated with vacuum disc that may or may not be herniated [67–77]. Moreover, entrapped gas formed within the degenerated vacuum disc is extruded into a herniated disc fragment, or the epidural and paravertebral space through tears in the annulus fibrosis causing radiculopathy [68, 69, 78–82]. Others also suggested that gas produced in the degenerative disc escaped through gaps in the degenerative endplate, forming a vertebral pneumatocyst [82–86]. Motion of the lumbar spine augments migration of discal gas into the epidural space by imposing excessive pressure on the intervertebral disc [69, 72, 75]. We believe that discal vacuum phenomenon that often is accompanied by disc height loss reduces the intervertebral volume space and increases the intradiscal pressure. This condition may lead, in some cases, to “vertical” herniation of the disc contends through the weakened part of the degenerated endplate causing SN. We also suggest that in these cases the presence of SN is a part of degenerative process of the lumbar spine and not only a radiographic marker being attributed by several factors such as genetics and environment [4, 13–16, 87]. As (1) vacuum phenomena and disc disease are associated with advanced age and as (2) we found that SN is significantly associated with disc disease (e.g., disc height loss and VP), this could explain the increased prevalence of SN with older age.

We believe that although SN is of multifactorial origin, it could also be a part of disc disease. Moreover, researchers should take into consideration the methods assessment and sampling, ethnicity, vascular disease, and the spine region when evaluating SNs.

There are some limitations of this study. No causal relationships between vacuum phenomenon and SN can be determined in this study. We believe that further larger-scale population studies based on CT images are needed in order to shed light on the pathogenesis of lumbar SNs.

## 5. Conclusions

Our results indicate that SN is a common phenomenon in the lumbar spine (44%), namely, the upper lumbar region (L1-2 to L3-4). Furthermore, disc degeneration (e.g., vacuum phenomenon and disc height loss), smoking habit, vertebral body length, and age increase the likelihood of lumbar SNs. However, the correlation between SNs and degeneration of the posterior spine elements (e.g., facet joint arthrosis) was not established.

## Figures and Tables

**Table 1:** The association between SNs and life-style variables.

**Variable**		**SN Prevalence (**%**)**	***X*** ^***2***^	**P value**
Smoking	No (n=113)	32.8	5.825	**0.02**
	Yes (n=67)	51.3		

Vascular disease	No (n=80)	38.8	1.891	0.178
	Yes (n=100)	49		

Heavy labor	No (n=130)	43.8	0.068	0.867
	Yes (n=50)	46		

**Table 2:** Vertebral body width and length (mean ± SD) for SNs and non-SNs groups by lumbar level.

**Variable**	**Non-SNs group** Mean ± SD (mm)	**SN group** Mean ± SD (mm)	**P value**
VW L1	36.8 ± 3.6	38 ± 3.4	**0.048**

VW L2	38.4 ± 3.4	39.4 ± 3.4	0.063

VW L3	40.4 ± 3.2	41.3 3.7	0.132

VW L4	42.3 ± 3	43.4 ± 3.9	**0.049**

VW L5	46.6 ± 3.9	47.8 ± 4.4	0.062

VL L1	27.5 ± 3	28.9 ± 2.9	**0.003**

VL L2	28.9 ± 2.9	29.9 ± 2.7	**0.022**

VL L3	30.5 ± 2.8	31.6 ± 2.6	**0.011**

VL L4	31 ± 2.7	31.9 ± 2.7	0.059

VL L5	31.6 ± 2.4	31.9 ± 2.6	0.377

VW: vertebral width; VL: vertebral length; SD: standard deviation.

## Data Availability

The data used to support the findings of this study are available from the corresponding author upon request.
